# Comparative genomic analysis of *Streptococcus suis* sequence type 105 and development of a PCR diagnostic tool

**DOI:** 10.1371/journal.pone.0324636

**Published:** 2025-05-20

**Authors:** Parichart Boueroy, Peechanika Chopjitt, Jednipit Borthong, Thidathip Wongsurawat, Piroon Jenjaroenpun, Rapeephan Duangjanchot, Bhanubong Saiboonjan, Rujirat Hatrongjit, Anusak Kerdsin

**Affiliations:** 1 Faculty of Public Health, Kasetsart University Chalermphrakiat Sakon Nakhon Province Campus, Sakon Nakhon, Thailand; 2 Division of Biology, Faculty of Science and Technology, Rajamangala University of Technology Thanyaburi, Pathumthani, Thailand; 3 Siriraj Long-read Laboratory, Division of Medical Bioinformatics, Department of Research and Development, Faculty of Medicine Siriraj Hospital, Mahidol University, Bangkok, Thailand; 4 Center for Innovation and Standard for Medical Technology and Physical Therapy, Faculty of Associated Medical Sciences, Khon Kaen University, Khon Kaen, Thailand; 5 Faculty of Science and Engineering, Kasetsart University Chalermphrakiat Sakon Nakhon Province Campus, Sakon Nakhon, Thailand; Cornell University, UNITED STATES OF AMERICA

## Abstract

*Streptococcus suis* serotype 14 is the second most prevalent serotype after serotype 2, and is highly prevalent in Southeast Asia. Among the serotype 14 strains, sequence type (ST) 105 is found in humans and pigs. We analysed the genome sequences of *S. suis* ST105 to identify unique sequences to develop a multiplex PCR (mPCR) -gel electrophoresis and mPCR-lateral flows trip (LFS) for epidemiological purposes. The ST105 genome was closely related to the ST1 genomes. All ST105 of Thai and Vietnamese strains were highly homologous. Of the 1818 genes found in all compared genomes, 36 unique sequences were detected only in the ST105 strain. Of these, two unique sequences encoding hypothetical proteins were selected as PCR targets. Only *S. suis* ST105 strains were positive for both mPCRs. mPCR-LFS had fewer complications, lower costs, and less time for testing, than those of mPCR-gel electrophoresis. This comparative genomic study demonstrates the usefulness of identifying unique sequences of ST105 *S. suis*. These unique sequences could be used to develop diagnostic or screening tools, such as PCR, for the detection of specific strains or clones for epidemiological purposes.

## Introduction

*Streptococcus suis* is a zoonotic pathogen that causes invasive infections in humans who are in close contact with pigs or pork by-products, or consume raw pork or pork by-product dishes [1]. Almost all *S. suis* serotype 2 infections are common in humans and swine [2]. *S. suis* serotype 14 is the second most prevalent serotype in Southeast Asia, particularly in Thailand [3,4]. Among the serotype 14 strains, sequence type (ST)105, which belongs to clonal complex (CC) 1, is prevalent in human and pig diseases, especially in Thailand and Vietnam [1,2,4]. According to the multilocus sequence typing (MLST) database, ST105 has also been reported in diseased pigs from the UK, in addition to humans (https://pubmlst.org/bigsdb?designation_operator1==&page=query&designation_value1=105&order=f_id&submit=1&db=pubmlst_ssuis_isolates&set_id=0&designation_field1=s_1_ST; accessed 1 June 2024).

Identification of ST105 relies on MLST and whole-genome sequencing [5,6]. However, these techniques are complex and not cost-effective. Simple *S. suis* CC PCR detection with low cost has been reported, but it cannot discriminate among STs within CCs [7]. In this study, we analysed the genome sequences of *S. suis* including STs 105, 237, and 1. Genomic comparisons revealed 36 unique sequences exclusive to the ST105 strains. Two unique genes were selected to design primers specific to ST105. Here, we describe a multiplex PCR (mPCR) method for identifying *S. suis* ST105 for epidemiological purposes.

## Materials & methods

### Bacterial strains

In total, 321 *S. suis* strains, divided into ST105 (n = 47) and non-ST105 (n = 274) groups, were included in the current study for mPCR evaluation (Table 1). In addition, other bacterial species were included to evaluate possible nonspecific reactions. These strains included *Streptococcus pneumoniae* ATCC33400, *S. gallolyticus* subsp. *gallolyticus* ATCC43143, *S. gallolyticus* subsp. *pasteurianus* ATCC43144, *S. intermedius* ATCC27335, *S. anginosus* ATCC33397, *S. pyogenes* SF370, *S. agalactiae* ATCC13813, *S. dysgalactiae* subsp. *equisimilis* CCUG36637, *S. dysgalactiae* subsp. *dysgalactiae* ATCC43078, *S. porcinus* ATCC43138, *S. bovis* ATCC33317, *S. oralis* ATCC35037, *S. mitis* ATCC6249, *S. sanguinis* ATCC10556, *S. gordonii* ATCC10558, *S. mutans* ATCC25175, *S. infantarius* subsp. *infantarius* ATCC BAA-102, *S. infantarius* subsp. *coli* ATCC27960, *S. constellatus* subsp. *pharyngis* CCUG46377, *S. constellatus* subsp. *constellatus* ATCC27823, *Enterococcus faecalis* ATCC29212, *Enterococcus faecium* ATCC10541, *Lactococcus plantarum* ATCC43199, *Leuconostoc lactis* ATCC19256, *Micrococcus luteus* ATCC10240, *Bacillus subtilis* ATCC6633, *Staphylococcus aureus* ATCC700698, *Haemophilus influenzae* ATCC10211, *Achromobacter xylosoxidans* ATCC27061, *Pseudomonas aeruginosa* ATCC9027, *Acinetobacter baumannii* ATCC19606, *Burkholderia cepacia* LMG0122, *Klebsiella pneumoniae* ATCC13883, *Escherichia coli* ATCC25922, *Enterobacter aerogenes* ATCC13048, and *Neisseria meningitidis* ATCC13077.

**Table 1:** *Streptococcus suis* used to evaluate mPCR of ST105 in this study.

Serotype	Clonal complex	Sequence types	Total (n = 321)	mPCR-gel electrohoresis -	mPCR-LFS
1 (n = 2)	1	**105**	**1**	**Positive**	**Positive**
		237	1	Negative	Negative
14 (n = 51)	1	1	1	Negative	Negative
		11	3	Negative	Negative
		**105**	**46**	**Positive**	**Positive**
		127	1	Negative	Negative
		237	1	Negative	Negative
2 (n = 197)	1	1	100	Negative	Negative
	25	25	20	Negative	Negative
		103	5	Negative	Negative
	28	28	12	Negative	Negative
	104	104	40	Negative	Negative
	233	233	40	Negative	Negative
		379	10	Negative	Negative
		1656	15	Negative	Negative
	1688	1688	5	Negative	Negative
4 (n = 7)	94	94	4	Negative	Negative
		1689	3	Negative	Negative
5 (n = 8)	none	181	1	Negative	Negative
	221/234	221	1	Negative	Negative
	none	235	2	Negative	Negative
		236	2	Negative	Negative
		483	1	Negative	Negative
		1197	1	Negative	Negative
9 (n = 1)	16	16	1	Negative	Negative
24 (n = 3)	221/234	221	2	Negative	Negative
		234	1	Negative	Negative
31 (n = 1)	221/234	221	1	Negative	Negative

### Whole-genome sequencing

All 11 genomes of *S. suis* serotype 14 which sequenced using Illumina MiSeq (Illumina, CA, USA) and Oxford Nanopore Technologies (ONT; Oxford, UK) platforms from a previous study [8], were used in the current study. Hybrid assemblies using ONT and Illumina data and genome sequence quality were performed using Unicycler v0.4.8 and QUAST v5.0.2, respectively [9,10]. The genome sequences were deposited in the NCBI Prokaryotic Genome Annotation Pipeline (PGAP v4.12) and default parameters were used for all software unless otherwise specified.

### Comparative genomic analysis

Comparative genomic analysis was performed using anvi’o v7 workflow [11]. This workflow identified gene clusters and single-copy genes in study genomes, including all 11 strains of serotype 14 including nine ST105 and each of STs 1 and 237 in this study, two Thai serotype 1 consist of ST105 strain 38828 (CP109941) and ST237 strain 35541 (CP109942); the Vietnamese serotype 14 ST105 strains EN314 (NZ_FIOV01000000), E34W (NZ_CDUM00000000), EN191 (NZ_FIOM00000000), and E11Q (NZ_CDWA00000000) [12]; and strain P1/7 (serotype 2-ST1; AM946016). The genome of strain P1/7 was used as the reference genome for comparison with all serotypes 14 and 1 genomes. The P1/7 strain is a well-characterised reference strain and belongs to CC1, similar to STs 1, 105 and 237. Therefore, the P1/7 genome was used as the reference for comparison with these genomes in the current study.

All genomes, in fasta format, were submitted for the analysis using the ‘anvi-run-workflow’ script. Genes were annotated using anvi-run-ncbi-cog. All genomes were added to a new anvi’o genomes storage using the ‘anvi-gen-genomes-storage’ application. The program ‘anvi-pan-genome’ ran the pan-genomic analysis on all the stored genomes using NCBI’s BLASTN tool. We used ‘anvi-import-misc-data’ to import additional metadata and ‘anvi-compute-genome-similarity’ to compute the average nucleotide identity (ANI) using the pyANI tool (https://github.com/widdowquinn/pyani). The pan-genome was visualised in anvi’o using the ‘anvi-display pan application. The whole pan-genome was divided into core and accessory bins based on gene cluster frequency. An UpSetR plot was generated using the UpSetR package in R program to visualise gene overlap across bacterial strains [13]. Specifically, gene lists from comparative genomic results were prepared and inputted into the UpSetR package to generate plots.

### Analysis of ST105 unique sequences

The local BLASTN [14] database was curated by compiling 8,090 *S. suis* genomes from two databases: 7,376 genomes from the NCBI database and 714 genomes from the PubMLST database. This database was used to assess the cross-reactivity of 36 unique sequences (S1 Table). To assess sequence similarities, the two unique sequences were used as queries (-query) and aligned against each genome as the subject (-subject). Alignments were performed using BLASTN with default parameters, except for the output format, which was set to tabular (-outfmt 6).

### Primer design

Of the 36 unique sequences present only in ST105, two sequences (hypothetical proteins 1 and 2) were selected based on their specificity, sequence length, and optimal criteria for primer design [15]. These two sequences were used as templates for primer design using Primer–BLAST (http://www.ncbi.nlm.nih.gov/tools/primer-blast/). The primer sequences are listed in Table 2.

**Table 2:** Primers and target genes used in the mPCR and mPCR-LFS.

Primer name	Sequence (5’-3’)	Size (bp)	Gene	Species/ ST	Purpose
CC1-F	CTTAAAGACCGTTATCAGACAACCT	2065	*srtBCD*	*S. suis* CC1	mPCR
CC1-R	ATCGAACTTGGAAACGAGCTTTCTC				
ST105-F1	CTTTTGCTGATTCCGATAAAACG	120	Hypothetical protein ID32567	ST105	mPCR
ST105-R1	AGTCTGTGAGTAATCCCAAACTG				
ST105-F2	TTGAAATCTTTCCTTATTCCCCATT	371	Hypothetical protein ID35001	ST105	mPCR
ST105-R2	CAGACCATAGAGACCGTCCAAG				
LFSST105-F1	FITC-CTTTTGCTGATTCCGATAAAACG	120	Hypothetical protein ID32567	ST105	mPCR-LFS
LFSST105-R1	Biotin-AGTCTGTGAGTAATCCCAAACTG				
LFSST105-F2	FITC-TTGAAATCTTTCCTTATTCCCCATT	371	Hypothetical protein ID35001	ST105	mPCR-LFS
LFSST105-R2	Digoxigenin-CAGACCATAGAGACCGTCCAAG				

### Multiplex PCR (mPCR)

All bacterial strains were cultured on sheep blood agar for 24 h at 37°C under ambient air conditions. Streptococci were incubated in 5% CO_2_. Pure colonies were selected from the sheep blood agar for DNA extraction. Genomic DNAs were extracted using a GF-1 Bacterial DNA Extraction Kit (Vivantis, Selangor Darul Ehsan, Malaysia) according to the manufacturer’s instructions.

The PCR reaction mixture contained 1 × PCRBIO HS Taq Mix Red (PCR Biosystems, London, UK) and 0.2 µM of each primer. The list of primers used for the PCR is presented in Table 2. The following PCR thermal profile was used: initial activation of DNA polymerase at 95°C for 3 min; 30 cycles of denaturation at 95°C for 20 s, primer annealing at 58°C for 30 s, and extension at 72°C for 1 min, followed by a final extension at 72°C for 5 min (T100 Thermal Cycler, Bio-Rad, CA, USA).

PCR products were analysed using gel electrophoresis (Mupid exU system, Japan) for 30 min on 2% agarose gels in 0.5 × TBE buffer. Gels were stained with ethidium bromide and visualised under ultraviolet light using GeneGenius Bioimaging System(SynGene, Cambridge, UK). The sizes of the PCR products (Table 2) were determined using comparisons with a molecular size standard (GeneRuler™ 100 bp Plus DNA ladder, Thermo Fisher Scientific, MA, USA).

### Lateral-flow strip (LFS)

LFS and reagents were purchased from K.Bio Sciences (Bangkok, Thailand) and used according to the manufacturer’s instructions. A strip was dipped into the PCR sample for at least 10–20 s, then the DNA running buffer (100 µL or three drops) was dropped on the application pad, and results were observed after 2–10 min. The development of both the test lines (T1, T2, or both) and the control (C) line indicated a positive result with amplified products, whereas the development of only the control line suggested a negative result.

### Detection limit

The detection limit of multiplex PCR was evaluated using *S. suis* ST105 strain 38828. Serial 10-fold dilutions were prepared from an original concentration of OD260, equivalent to 72 ng/µL. The detection limit was determined as the highest dilution concentration that showed a positive result. This evaluation was performed in triplicates.

### Ethics statement

This study does not involve either human or animal study. There is no requirement for approval by the relevant institutional review board or ethics committee because the stored bacterial isolates used in this study were not considered to constitute human or animal subject research because no human or animal specimens or data were used.

### Accession number

The genome sequences of the 11 *S. suis* serotype 14 strains were deposited in the NCBI GenBank under Bioproject accession number PRJNA986293.

## Results and discussion

### Comparative genomic analysis

**Fig 1:**
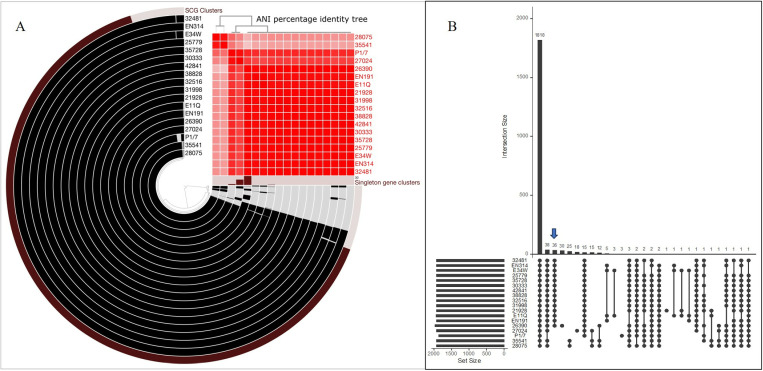
Comparative genomic analysis representation based on anvi’o software. (A) Pan-genome reconstructed with 15 genomes of *S. suis* serotype 14, two genomes of serotype 1 (38828 and 35541), and a reference genome of serotype 2 (P1/7) strains. Each ring in the graph represents an individual *S. suis* genome, and each ray corresponds to a given gene homolog. The four inner layers are in black to designate gene clusters in that genome or translucent if the gene cluster is absent. The ANI result was visualized in the heatmap tree. (B) UpSetR shows the number of genes that are shared and unique in *S. suis* serotypes 14, 1, and 2 genomes from pangenome analysis based on anvi’o software with 15 genomes of *S. suis* serotype 14, two genomes of serotype 1 (38828 and 35541), and reference genome of serotype 2 (P1/7) strains. ANI, average nucleotide identity. The blue arrow indicates 36 unique sequences identified in ST105.

As shown in Fig 1, we compared all serotype 14-ST105 genomes of Thai (21928, 25779, 26390, 31998, 32481, 32516, 35728, 30333, and 42841), Vietnamese (EN314, E34W, E11Q, and EN191), Thai serotype 1-ST105 (38828), Thai serotype 1-ST237 (35541), Thai serotype 14-ST237 (28075), Thai serotype 14-ST1 (27024), and serotype 2-ST1 (P1/7) as reference genomes. Theses strains belong to CC1. As shown in Fig 1A, the ST105 genome was closely related to the ST1 genome and quite different from the ST237 genome. Of the 1818 genes found in all comparative genomes in this study, 38 were not present in reference P1/7 (Fig 1B). Fig 1B shows unique genes present only in ST105 (n = 36), ST237 (n = 25), STs 1 and 105 (n = 15), STs 105 and 237 (n = 38), Vietnamese ST105 (n = 5), and Thai STs 105, 237, and ST1 (n = 2). Notably, the Thai serotype14-ST105 genome comparison between the three outbreak strains (31998, 32481, and 32516) [4,16] and six sporadic strains (21928, 25779, 26390, 30333, 35728, and 42841) showed no differences in the genomes, and no specific or unique genes were present in the outbreak strains, as shown in Fig 1. All outbreak and sporadic strains had highly homologous genomes.

The ST105 in both Thai and Vietnamese strains contained 36 unique genes including DNA recombinase, DNA invertase, DNA topoisomerase IA (TopA), peptide pheromone, LytR/AlgR family DNA-binding response regulator, thioredoxin (TrxA), sensor histidine kinase YesM, ABC-type bacteriocin/lantibiotic exporters, metabolic enzymes, membrane proteases, multidrug resistance efflux pump EmrA, Mg^2+^ and Co^2+^ transporter CorA, and abortive infection bacteriophage resistance protein (AbiF) and abortive phage infection protein AbiEi. However, the two genes absent from the Vietnamese ST105 genome encoded acyltransferase family proteins and hypothetical proteins. The details of these unique genes are listed in S1 Table. In addition, the functional roles of these unique genes require further characterization.

A previous study demonstrated that the Thai serotype 1-ST105 strain 38828 was highly homologous to Vietnamese ST105 strains, including EN314, E34W, E11Q, and EN191 [12]. The current study showed that all ST105 (serotypes 1 and 14) of Thai and Vietnamese strains were also highly homologous, suggesting they have a common ancestor and may circulate in the Thailand-Lao PDR-Vietnam area [16]. In addition, we found a very high homology of the ST237 genome between Thai human-derived serotype 14 strain 28075 and Thai porcine-derived serotype 1 strain 35541. Although different serotypes, serotypes 1 and 14 have almost identical capsule loci [17,18]. This indicates that pigs are a reservoir of ST237-pathogenic strains in humans and clinically asymptomatic pigs contain pathogenic human strains, as previously mentioned [19–21].

### Development of mPCR-based identify *S. suis* ST105

*S. suis* ST105 belongs to CC1 [2]. It was first described in Vietnam in 2004 from an adult case of meningitis [22]. Later, a cluster of ST105 strains belonging to serotype 14 was reported in Thailand [3]. In 2023, serotype 1-ST105 was reported in Thailand [12]. ST105 is also found in diseased pigs in the UK, highlighting its clinical and epidemiological importance.

We developed an mPCR assay to identify ST105 in *S. suis*, which is clinically important and causes invasive infections in humans and pigs. Based on the comparative genomes of the ST105 strains with the reference ST1 strains (Fig 1), the ST105 strains from both Thai and Vietnamese samples contained 36 unique genes (S1 Table). Among these 36 unique genes, we performed a local BLASTN analysis against all *S. suis* genomes (n = 8,090) to evaluate cross-reactivity. Among these 36 unique sequences, two unique sequences—hypothetical proteins ID35001 and ID32567 revealed high specificity from local BLASTN analysis (S2 Table).

Among the 8,090 *S. suis* genomes, two genomes had sequences homologous to the hypothetical protein ID32567 gene only, whereas only 187 genomes contained sequences homologous to the hypothetical protein gene ID-35001 (S2 Table). No genomes were positive for both target sequences, except 10 genomes belonging to ST105 (S2 Table). These two unique sequences were thus deemed appropriate for detecting ST105. Consequently, they were chosen for primer design and developed for mPCR. Additionally, primers specific to *S. suis* CC1 were included in the mPCR -gel electrophoresis (Table 2) to enhance specificity for *S. suis* ST105. Only ST105 would be positive for three targets (CC1, hypothetical protein genes ID35001, and ID35267).

**Fig 2:**
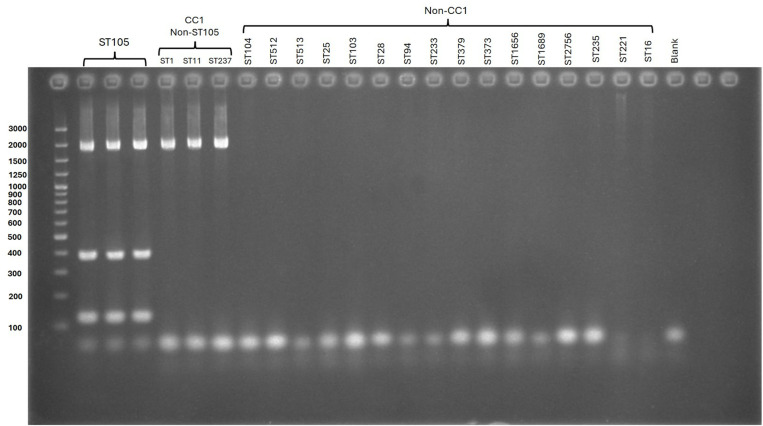
Agarose gel electrophoresis of PCR-amplified products from mPCR of *S. suis* ST105; lane 1 = ST105 (strain 32481), lane 2 = ST105 (strain 38828), lane 3 = ST105 (strain 31998), lane 4 = ST1 (strain P1/7), lane 5 = ST11 (strain 32599), lane 6 = ST237 (strain 35541), lane 7 = ST104 (strain 24525), lane 8 = ST512 (strain 39971), lane 9 = ST513 (strain 40133), lane 10 = ST25 (strain 30190), lane 11 = ST103 (strain 25781), lane 12 = ST28 (strain 23164), lane 13 = ST94 (strain 36054), lane 14 = ST233 (strain 35688), lane 15 = ST379 (strain 36602), lane 16 = ST373 (strain STC2826), lane 17 = ST1656 (strain STC78), lane 18 = ST1689 (strain 34533), lane 19 = ST2756 (strain MN33), lane 20 = ST235 (strain 32563), lane 21 = ST221 (strain 33329), lane 22 = ST16 (strain 45624), and lane 23 = no template control.

**Fig 3:**
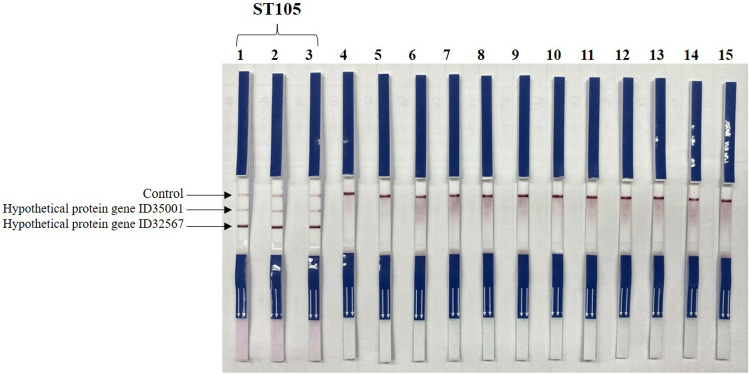
Lateral flow strip detection of PCR-amplified products from mPCR of *S. suis* ST105. From left to right, strip 1 = ST105 (strain 32481), strip 2 = ST105 (strain 38828), strip 3 = ST105 (strain 31998), strip 4 = ST1 (strain P1/7), strip 5 = ST1 (strain 26154), strip 6 = ST11 (strain 32599), strip 7 = ST237 (strain 35541), strip 8 = ST25 (strain 30190), strip 9 = ST28 (strain 23164), strip 10 = ST104 (strain 24525), strip 11 = ST233 (strain 35688), strip 12 = ST1656 (strain STC78), strip 13 = ST221 (strain 33329), strip 14 = ST94 (strain 36054), strip 15 = no template control. mPCR, multiplex PCR.

mPCR developed in the current study was applied to *S. suis* and other bacteria. As shown in Table 1, only *S. suis* ST105 strains tested positive, displaying a *S. suis* CC1 band at 2065 bp and two ST105-specific bands at 120 and 371 bp. In contrast, other *S. suis* STs in CC1 showed only the 2065 bp *S. suis* CC1 band. Non-CC1 *S. suis* and non-*S. suis* bacteria tested negative for *S. suis* CC1 and ST105 (Fig 2). This indicated that mPCR can be used to detect ST105 in *S. suis* CC1. To facilitate easy interpretation, save time, and reduce the cost of gel electrophoresis, we labeled the mPCR-LFS primers with biotin, digoxigenin, and FITC (Table 2). Fig 3 revealed ST105 positive bands on the strip, whereas non-ST105 *S. suis* strains and other bacteria tested in this study showed negative results. However, we strongly recommend identifying *S. suis* and its CCs, especially CC1, before applying mPCR to increase accuracy and reliability for ST105 identification (ST105 belong to CC1) [7,23]. (S2 Table).

In the present study, we developed two types of mPCR: (1) mPCR-gel electrophoresis and (2) mPCR-LFS. The mPCR-LFS demonstrates better cost and time effectiveness than those of mPCR-gel electrophoresis. Excluding DNA extraction, the cost per sample for mPCR-LFS is approximately 2.25–2.45 USD, while for mPCR-gel electrophoresis, it is 2.75–3.15 USD. The running time for mPCR-LFS was approximately 75–90 min, whereas that for mPCR-gel electrophoresis was approximately 120–130 min. PCR-LFS has been successfully used to detect several pathogenic bacteria, including *Bacillus anthracis*, *Yersinia pestis*, and *Salmonella* [24–26]. The detection limits for these pathogens in those previous studies were 1–5 pg of genomic DNA. In contrast, our mPCR-LFS assay has a lower detection limit of 72 fg, whereas the analytical detection limit of mPCR-gel electrophoresis is 720 fg (0.72 pg) of *S. suis* genomic DNA. This finding suggests that mPCR-LFS is more sensitive than mPCR-gel electrophoresis.

Since its development, MLST has been considered the gold standard for determining the structure of *S. suis* populations [5,6]. This aids the identification of pathogenic *S. suis* isolates, particularly when combined with serotyping, as demonstrated in previous studies [27]. However, MLST is complicated, time-consuming, costly for resource-limited laboratories, and lacks high throughput for large numbers of isolates. In 2016, mPCR was used to identify *S. suis* CCs associated with human infections [7]. Unfortunately, mPCR could not discriminate between STs within CCs. This is the first study to demonstrate that mPCR can identify ST105 of CC1 without cross-reactivity with other STs in CC1. This indicates that mPCR can be used as a surveillance tool for screening ST105 in endemic areas, such as Thailand and Vietnam.

## Conclusions

We demonstrated the usefulness of genomic comparisons for identifying genetic markers of *S. suis* ST105. Of the 36 unique sequences identified in ST105, only two unique sequences (hypothetical proteins) could be applied for detecting *S. suis* ST105 among CC1 strains tested. These markers can be used to develop diagnostic or screening tools, such as mPCR. The mPCR-LFS method demonstrated in this study was highly sensitive, rapid, uncomplicated, and cost-effective. The mPCR method in this study could be used for the epidemiological purposes of *S. suis* ST105 monitoring or screening of *S. suis* ST105 from a large number of isolates.

## Supporting information

S1 Table Unique genes present in only *Streptococcus suis* serotype 14 ST105. (XLSX)

S2 Table BLASTN analysis of two selected unique sequences encoding hypothetical proteins ID32567 and ID35001 against 8,090 *Streptococcus suis* genomes. (XLSX)

S3 Table Whole-genome sequencing parameters of 11 *Streptococcus suis* serotype 14. (DOCX)

S1 Figure Original uncropped and unadjusted gel electrophoresis image. (TIF)
