# Supplementary material for: Comparative genomic analysis of Streptococcus suis sequence type 105 and development of a PCR diagnostic tool
Source: PLoS One. 2025 May 20;20(5):e0324636. doi: 10.1371/journal.pone.0324636 (PMC12091807; doi:10.1371/journal.pone.0324636)
Supplement: S3 Table — (DOCX) [file pone.0324636.s003.docx]

**S3 Table**. Whole-genome sequencing parameters of 11 *Streptococcus suis* serotype 14

| **Strain no.** |  | **Illumina data** | | | | | **Nanopore data** | | | | | | **Assembly data by Unicycler** | | | |
| --- | --- | --- | --- | --- | --- | --- | --- | --- | --- | --- | --- | --- | --- | --- | --- | --- |
|  | **Total size (bp)** | **No. of reads** | **N50** | **Sequence length** | **No. of contig** | **GC content (%)** | **No. of reads** | **Mean read quality** | **Mean read length** | **N50** | **GC content (%)** | **No. of contig** | **Total size (bp)** | **No. of contig** | **GC content (%)** | **N50** |
| 21928 | 2055716 | 279222 | 244 | 15-244 | 41 | 41.19 | 159873 | 14.0 | 3827.6 | 6835 | 41.2 | 3 | 2074659 | 3 | 41.24 | 1987823 |
| 25779 | 2054513 | 211533 | 244 | 15-244 | 41 | 41.19 | 105947 | 14.0 | 4350.6 | 8022 | 41.2 | 1 | 2074837 | 1 | 41.24 | 2074837 |
| 26390 | 2106611 | 403761 | 244 | 15-244 | 25 | 41.07 | 138862 | 14.0 | 4240.2 | 7849 | 41.1 | 1 | 2127224 | 1 | 41.12 | 2127224 |
| 27024 | 2048290 | 618184 | 244 | 15-244 | 44 | 41.15 | 54766 | 14.1 | 2927.5 | 5817 | 41.2 | 1 | 2068913 | 1 | 41.20 | 2068913 |
| 28075 | 2073202 | 363621 | 244 | 15-244 | 34 | 41.10 | 121812 | 14.0 | 4017.6 | 7375 | 41.2 | 1 | 2096230 | 1 | 41.15 | 2096230 |
| 30333 | 2053980 | 343672 | 244 | 15-244 | 32 | 41.19 | 158402 | 14.0 | 4194.9 | 7521 | 41.2 | 1 | 2074522 | 1 | 41.24 | 2074522 |
| 31998 | 2053037 | 476987 | 244 | 15-244 | 32 | 41.18 | 69295 | 14.1 | 3697.2 | 7028 | 41.2 | 1 | 2074568 | 1 | 41.24 | 2074568 |
| 32481 | 2053275 | 422180 | 244 | 15-244 | 38 | 41.19 | 380007 | 14.6 | 4114.5 | 7230 | 41.2 | 2 | 2072798 | 2 | 41.24 | 1985962 |
| 32516 | 2053323 | 370533 | 244 | 15-244 | 33 | 41.18 | 80737 | 14.1 | 2859.4 | 5401 | 41.1 | 1 | 2074350 | 1 | 41.24 | 2074350 |
| 35728 | 2053384 | 354131 | 244 | 15-244 | 36 | 41.18 | 127034 | 14.0 | 4535.1 | 8305 | 41.2 | 1 | 2074809 | 1 | 41.24 | 2074809 |
| 42841 | 2054600 | 267722 | 244 | 17-244 | 37 | 41.19 | 94164 | 14.1 | 3000.3 | 5759 | 41.2 | 1 | 2074552 | 1 | 41.24 | 2074552 |
