# Supplementary figures and images for: Comparative genomic analysis of Streptococcus suis sequence type 105 and development of a PCR diagnostic tool
Source: PLoS One. 2025 May 20;20(5):e0324636. doi: 10.1371/journal.pone.0324636 (PMC12091807; doi:10.1371/journal.pone.0324636)

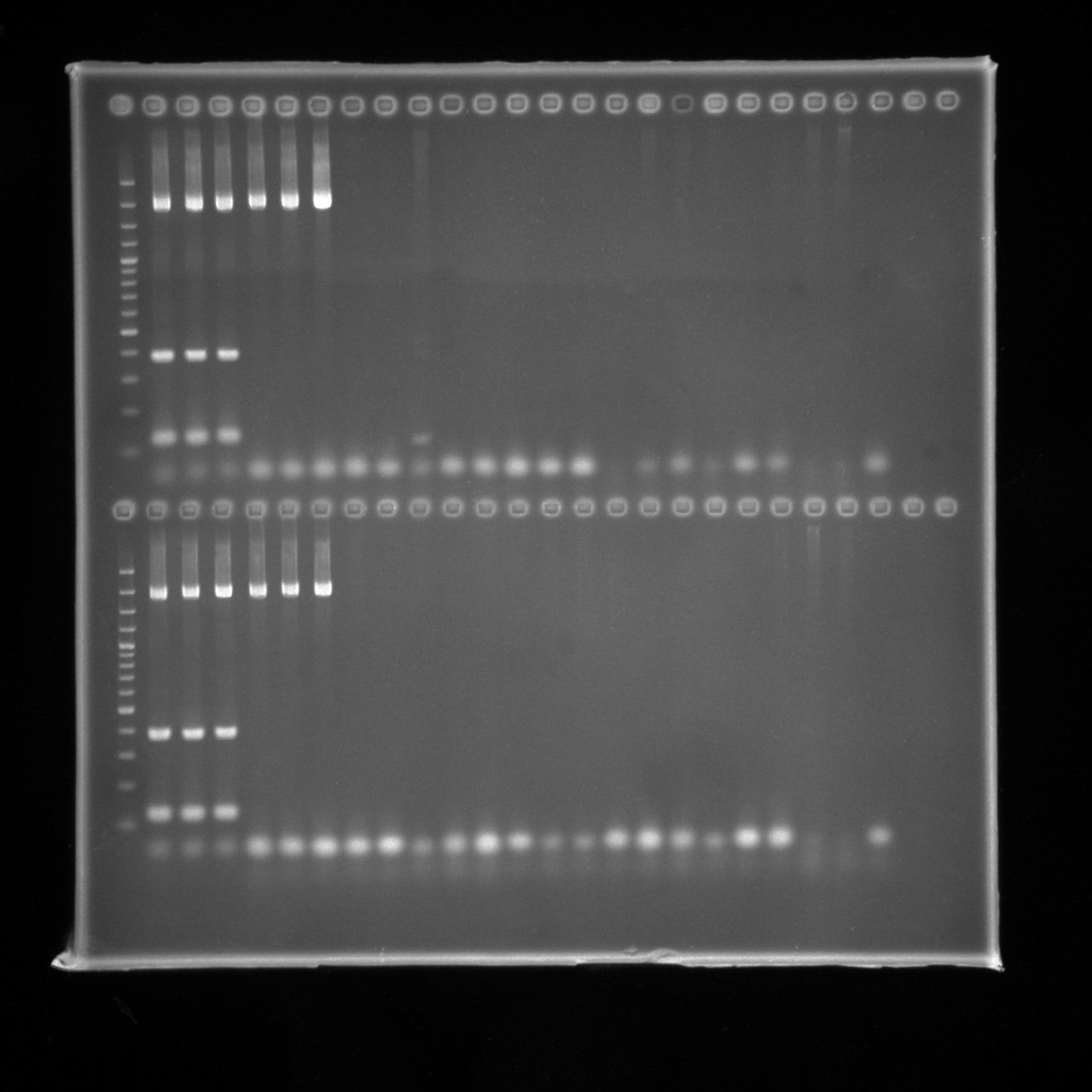

Supplement: S1 Figure — (TIF) [file pone.0324636.s004.tif]
